# Uncontrolled asthma in school-aged children—a nationwide specialist care study

**DOI:** 10.1016/j.jacig.2024.100227

**Published:** 2024-02-13

**Authors:** Caroline Stridsman, Øyvind Martinsen, Stina Selberg, Maria Ödling, Jon R. Konradsen

**Affiliations:** a Department of Public Health and Clinical Medicine, Division of Medicine/OLIN Unit, Umeå University, Umeå, Sweden; b Department of Women’s and Children’s Health, Karolinska Institutet, Stockholm, Sweden; d Department of Clinical Science and Education, Södersjukhuset, Karolinska Institutet, Stockholm, Sweden; c Astrid Lindgren’s Children’s Hospital, Karolinska University Hospital, Stockholm, Sweden

**Keywords:** Asthma, children, asthma management, asthma phenotypes, school-aged asthma, severe asthma, asthma control, asthma treatment, undertreated asthma, pulmonary function, obesity, exacerbations, uncontrolled asthma

## Abstract

**Background:**

Uncontrolled asthma (UCA) is different from severe asthma and can be identified in children across all ranges of prescribed treatment.

**Objective:**

Our aim was to characterize uncontrolled childhood asthma in pediatric specialist care.

**Methods:**

We performed a nationwide cross-sectional study of 5497 children (aged 6-17 years) with asthma who were treated by pediatricians at outpatient clinics during 2019 and registered in the Swedish National Airway Register. UCA was defined as an Asthma Control Test score of 19 or lower and/or 2 or more exacerbations in the past year and/or an FEV_1_ value less than 80% predicted. Treatment was categorized from step 1 to step 5 according to the Global Initiative for Asthma.

**Results:**

UCA was identified in 1690 children (31%), of whom 64% had an Asthma Control Test score of 19 or lower, 20% had recurrent exacerbations, and 31% had an FEV_1_ value less than 80% predicted. UCA was associated with female sex (odds ratio [OR] = 1.29 [95% CI = 1.15-1.45]), older age (OR = 1.02 [95% CI = 1.00-1.04]), obesity (OR = 1.43 [95% CI = 1.12-1.83]), and more treatment using steps 1 and 2 as a reference (step 3, OR = 1.28 [95% CI = 1.12-1.46]); steps 4-5, OR = 1.32 [95% CI = 1.10-1.57]). UCA in children prescribed treatment steps 1 and 2 (group UCA1-2) occurred in 28% of all children at this treatment step (n = 887). Children in group UCA1-2 had exacerbations more frequently than did those children with UCA who were prescribed steps 4 and 5 treatment (24% vs 15% [*P* = .001]).

**Conclusion:**

UCA was common and associated with female sex, increasing age, obesity, and higher Global Initiative for Asthma treatment step. Surprisingly, UCA was also common in children prescribed less than the maximum treatment, and those children could be considered undertreated patients.

Asthma remains the most common chronic disease in childhood, with a worldwide prevalence of around 10% in children and adolescents.^1^ The critical outcome of asthma management is improved asthma control,^2^, ^3^, ^4^ defined as the extent to which the manifestations of asthma have been reduced or removed by treatment.^3^ The manifestations of childhood uncontrolled asthma (UCA) include poor symptom control, recurrent exacerbations, and low lung function.^2^^,^^5^

Many children with asthma can obtain asthma control through avoidance of triggering factors and/or with inhaled β_2_-agonists and inhaled corticosteroids (ICSs), according to steps 1 and 2 in the Global Initiative for Asthma (GINA) guidelines.^6^ Nevertheless, worldwide data from questionnaire-based epidemiologic studies have shown that the prevalence of UCA in children is more than 50%.^7^ However, population-based clinical data on childhood UCA in a European setting are sparse.

UCA is not synonymous with severe asthma, as the latter is diagnosed either in children who need GINA steps 4 and 5 treatment to obtain control or in children with UCA despite maximum treatment.^2^ From these definitions, it is clear that UCA also can occur in children who are not prescribed steps 4 and 5 treatment. However, to our knowledge, the prevalence and characteristics of UCA in children prescribed less than the maximum treatment have never been investigated in a large population-based asthma cohort using established definitions of asthma control.

We hypothesized that childhood UCA is prevalent across all ranges of prescribed treatment (from GINA step 1 through GINA step 5) in a Swedish specialist care setting and that patient characteristics (age, sex, body mass index [BMI], and allergy) and manifestations of UCA (poor symptom control, recurrent exacerbations, and/or low lung function) differ between children prescribed GINA steps 1 and 2 treatment and children prescribed steps 4 and 5 treatment. Our primary aim was to determine the prevalence, characteristics, and prescribed treatment associated with UCA by using data from a sizeable real-life cohort of school-aged children treated by pediatricians in Sweden. Second, we aimed to explore whether characteristics and manifestations of UCA in children prescribed GINA steps 1 and 2 treatment differ from those in children prescribed GINA steps 4 and 5 treatment.

## Methods

Ours was a cross-sectional cohort study using data from the Swedish National Airway Register (SNAR), a quality register that includes data on children with asthma who were referred to and treated by pediatricians at outpatient clinics in Sweden.^8^ The SNAR contains detailed information regarding symptoms, pulmonary function, and medications and thus constitutes a unique platform for analysis of asthma control, severity, and treatment. The current study complied with the Declaration of Helsinki and was approved by the Swedish Ethical Review Authority (2019-04915). Children with asthma aged 6 to 17 years were identified from pediatric specialist care recordings in the SNAR that were made from January 1 to December 31, 2019 (n = 7338). Children younger than 6 years were not included, as pulmonary function measurements were not available in the vast majority of those cases. Registrations with missing data on asthma medication were excluded (n = 1841), leaving a total of 5497 children who were included in the current study. Children without reported medication were younger, had higher Asthma Control Test (ACT) scores, had higher FEV_1_% predicted values, and reported fewer exacerbations than did children with reported medication.

### Definitions

UCA was defined in patients with an ACT score of 19 or lower and/or 2 or more reported exacerbations in the past year and/or an FEV_1_ value less than 80% of predicted according to the American Thoracic Society/European Respiratory Society guidelines.^2^

Treatment reported to the SNAR includes short-acting β_2_-agonists (SABAs), short-acting muscarinic antagonists, ICSs, long-acting β_2_-agonists (LABAs), leukotriene receptor antagonists (LTRAs), and long-acting muscarinic antagonists, as well as use of the Airsonett medical device (Airsonett AB, Ängelholm, Sweden) and biologic treatments such as omalizumab, dupilumab, and mepolizumab. The use of asthma medications was categorized according to the 2018 update of the GINA guidelines as follows: step 1, a SABA or LABA, as needed; step 2, single-drug therapy with an ICS or LTRA as a controller medication; step 3, treatment with 2 controller medications (ICS + LABA or ICS + LTRA or LTRA + LABA); step 4: treatment with at least 3 controller medications (ICS, LABA, long-acting muscarinic antagonist, and/or LTRA); and step 5: biologic treatments (n=34) and/or treatment with temperature laminar airflow (n = 42), with the latter being an additional step 5 option according to the national Swedish guidelines.^9^

*Allergy, exacerbations, treatment plan, and patient education* were defined as physician-reported, affirmative, or numeric responses to the following questions in the SNAR: “Does the patient have an allergy diagnosis?” “How many exacerbations has the patient had in the previous 12 months?” “Has the patient been provided with a written treatment plan?” and “Has the patient received structured asthma education the past 5 years (introduction to asthma, self-management, risk and aggravating factors, and inhalation technique)?”

BMI was calculated and age-dependent cutoffs for normal weight, overweight, and obesity provided by the International Obseity Task Force were used.^10^

*Lung function* was assessed through FEV_1_ value as a percentage of the predicted value (FEV_1_%). Postbronchodilator results with the Solymar reference values were used.^11^ FEV_1_% of predicted was used as a continuous variable and also categorized according to whether the patient’s FEV_1_ value was less than 80% of the predicted value.

### Data availability

The data set is held and managed by the Region Norrbotten, Luleå, Sweden. Relevant anonymized data are available on reasonable request following approval from the Swedish Ethical Review Authority.

### Statistical analysis

All statistical analyses were conducted using IBM SPSS Statistics (version 27 [IBM Inc, Armonk, NY]). To assess differences between groups, independent sample *t* tests, ANOVA, or chi-square tests were used. *P* values less than .05 were considered statistically significant. Odds ratios (ORs) and 95% CIs were estimated in bivariate and multivariate logistic regression analyses. The dependent variables were UCA and UCA in children prescribed GINA steps 1 and 2 treatment. All models were adjusted for sex, age, BMI categories, and allergy and also stratified by sex and age.

## Results

### Demographic characteristics

In this nationwide register–based cohort of school-aged children with asthma treated by pediatricians (n = 5497), their mean age was 11.2 years, and the majority were boys (62.0%). The mean ACT score was lower in females than in males, and a larger proportion of females had an ACT score of 19 or lower, whereas males had a lower FEV_1_% value than females did (Table I). Dropout analysis revealed that children with missing ACT scores and FEV_1_ values were younger, had a lower BMI, and were more frequently prescribed steps 1 and 2 treatment than children in whom these data were reported (see Table E1 in the Online Repository at www.jaci-global.org). In addition, children with missing data from the ACT had fewer exacerbations.

**Table I:** Demographic characteristics

Characteristic	All	Girls	Boys	*P* value	Aged 6-11 y	Aged 12-17 y	*P* value	Controlled asthma	UCA	*P* value
	n = 5497	n = 2091	n = 3406		n = 2994	n = 2503		n = 3807	n = 1690	
Percentage of entire cohort		38.0	62.0		54.5	45.5		69.3	30.7	
Female sex, no. (%)	2091 (38.0)				1106 (36.9)	985 (39.4)	.067	1379 (36.2)	712 (41.1)	**<.001**
Age (y), mean (SD)	11.2 (3.2)	11.3 (3.3)	11.1 (3.2)	**.012**				11.1 (3.3)	11.4 (3.1)	**<.001**
BMI (kg/m^2^), mean (SD)∗	19.3 (3.9)	19.5 (3.9)	19.2 (3.9)	**.019**	17.7 (3.0)	21.1 (4.0)	**<.001**	19.2 (3.8)	19.6 (4.1)	**<.001**
Normal weight, no. (%)	3952 (76.9)	1506 (77.4)	2446 (76.6)		2135 (77.9)	1817 (76.8)		2715 (77.9)	1237 (74.8)	
Overweight, no. (%)	898 (17.5)	332 (17.1)	566 (17.7)		481 (17.4)	417 (17.6)		597 (17.1)	301 (18.2)	
Obesity, no. (%)	288 (5.6)	108 (5.5)	566 (5.6)	.811	155 (5.6)	133 (5.6)	.969	173 (5.0)	115 (7.0)	**.007**
Allergy, no. (%)	2758 (50.2)	985 (47.1)	1773 (52.1)	**<.001**	1219 (40.7)	1539 (61.5)	**<.001**	1882 (49.4)	876 (51.8)	.101
ACT score, mean (SD)†	21.3 (3.6)	20.7 (3.9)	21.7 (3.4)	**<.001**	21.6 (3.8)	21.0 (3.4)	**<.001**	23.0 (1.9)	18.3 (4.0)	**<.001**
ACT score ≤ 19, no. (%)	1084 (24.9)	506 (31.0)	578 (21.4)	**<.001**	514 (23.1)	570 (27.0)	**.003**	0 (0.0)	1084 (68.2)	NA
≥2 exacerbations, no. (%)	337 (6.1)	126 (6.0)	211 (6.2)	.800	196 (6.5)	141 (5.6)	.160	0 (0.0)	337 (19.9)	NA
FEV_1_% predicted, mean (SD)‡	91.3 (12.5)	92.1 (12.6)	90.8 (12.3)	**.001**	91.1 (11.9)	91.6 (13.1)	.142	94.9 (9.8)	85.6 (14.2)	**<.001**
FEV_1_ < 80% predicted, no. (%)	532 (15.5)	191 (14.4)	341 (16.2)	.173	272 (15.1)	260 (15.9)	.513	0 (0.0)	532 (40.7)	NA
UCA, no. (%)§	1690 (30.7)	712 (34.1)	978 (28.7)	**<.001**	859 (28.7)	831 (33.2)	**<.001**			
Treatment plan, no. (%)	2223 (40.4)	818 (39.1)	1405 (41.3)	.118	1181 (39.4)	1042 (41.6)	.100	1439 (37.8)	784 (46.4)	**<.001**
Asthma education, no. (%)	3679 (66.9)	1384 (66.2)	2295 (67.4)	.362	1871 (62.5)	1808 (72.2)	**<.001**	2438 (64.0)	1241 (73.4)	**<.001**

Percentages and mean values calculated for those with complete data regarding BMI, ACT score, and FEV_1_ value. Boldface indicates statistical significance.

*NA,* Statistical calculation is not applicable, as the observed differences reflect the inclusion criteria of the groups compared.

∗ BMI missing for 359 children.

† ACT score missing for 1162 children.

‡ FEV_1_ value missing for 2062 children.

§ UCA defined as an ACT score of 19 or lower and/or 2 or more exacerbations in the past year and/or an FEV_1_ value less than 80% predicted.

### Asthma control

**Fig 1:**
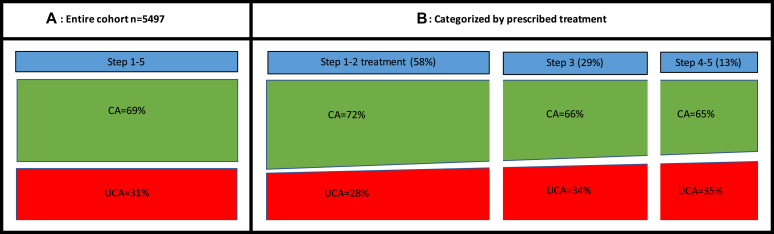
Proportion of children with controlled asthma (CA) and UCA among 5497 school-aged children with asthma (**A**) and categorized by prescribed treatment (**B**).

In total, 1690 children (31%) had UCA (Fig 1, *A* and Table I). The children with UCA were older, more frequently female, and obese than those with controlled asthma. Furthermore, the children with UCA received asthma management education and written action plans more often (Table I). In adjusted regression analysis, female sex, older age, obesity, and more prescribed treatment were associated with UCA. Stratification by sex showed that older age was associated with UCA in females, whereas associations with obesity and more prescribed treatment were found in males only. Stratification by age revealed that UCA was related to obesity and prescribed treatment in younger children and to females in adolescence (Table II).

**Table II:** Bivariate and multivariate logistic regression models of factors associated with UCA with controlled asthma as a reference and UCA with steps 1 and 2 treatment with controlled asthma steps 1 and 2 treatment as reference

Characteristic	All		Girls	Boys	Aged 6-11 y	Aged 12-17 y
	Crude OR (95% CI)	Adjusted OR (95% CI)	Adjusted OR (95% CI)	Adjusted OR (95% CI)	Adjusted OR (95% CI)	Adjusted OR (95% CI)
Model A: UCA (controlled asthma as a reference)						
Female sex	**1.28 (1.14-1.44)**	**1.29 (1.15-1.45)**			0.94 (0.80-1.11)	**1.81 (1.53-2.15)**
Older age	**1.04 (1.02-1.05)**	**1.02 (1.00-1.04)**	**1.09 (1.06-1.12)**	0.98 (0.95-1.00)		
Normal weight	REF	REF	REF	REF	REF	REF
Overweight	1.11 (0.95-1.29)	1.10 (0.95-1.29)	1.10 (0.86-1.42)	1.12 (0.92-1.37)	1.16 (0.93-1.43)	1.07 (0.85-1.34)
Obesity	**1.46 (1.14-1.86)**	**1.43 (1.12-1.83)**	1.19 (0.79-1.79)	**1.64 (1.20-2.24)**	**1.59 (1.13-2.22)**	1.33 (0.92-1.91)
Treatment steps 1-2	REF	REF	REF	REF	REF	REF
Treatment step 3	**1.34 (1.18-1.52)**	**1.28 (1.12-1.46)**	1.09 (0.89-1.35)	**1.43 (1.20-1.69)**	**1.38 (1.15-1.65)**	1.19 (0.98-1.44)
Treatment steps 4-5	**1.36 (1.15-1.62)**	**1.32 (1.10-1.57)**	1.29 (0.96-1.71)	**1.37 (1.09-1.72)**	**1.53 (1.16-2.01)**	1.20 (0.95-1.51)
Model B: UCA steps 1 and 2 (controlled asthma steps 1-2 as a reference)						
Female sex	**1.32 (1.13-1.55)**	**1.32(1.13-1.55)**			0.94 (0.76-1.16)	**2.18 (1.69-2.80)**
Older age	**1.05 (1.03-1.08)**	**1.04 (1.02-1.07)**	**1.12 (1.08-1.16)**	0.99 (0.96-1.02)		
Normal weight	REF	REF	REF	REF	REF	REF
Overweight	1.13 (0.92-1.40)	1.13 (0.91-1.39)	1.18 (0.85-1.64)	1.13 (0.86-1.49)	1.18 (0.90-1.54)	1.09 (0.78-1.53)
Obesity	1.25 (0.88-1.78)	1.24 (0.87-1.77)	1.03 (0.58-1.83)	1.48 (0.94-2.34)	1.42 (0.90-2.22)	1.06 (0.59-1.91)

UCA defined as an ACT score of 19 or lower and/or 2 or more exacerbations in the past year and/or an FEV_1_ value less than 80% predicted. Normal weight reference to overweigh and obesity. Age was entered as a continuous variable. Boldface indicates statistical significance.

*REF*, Reference.

### Manifestations of UCA

**Fig 2:**
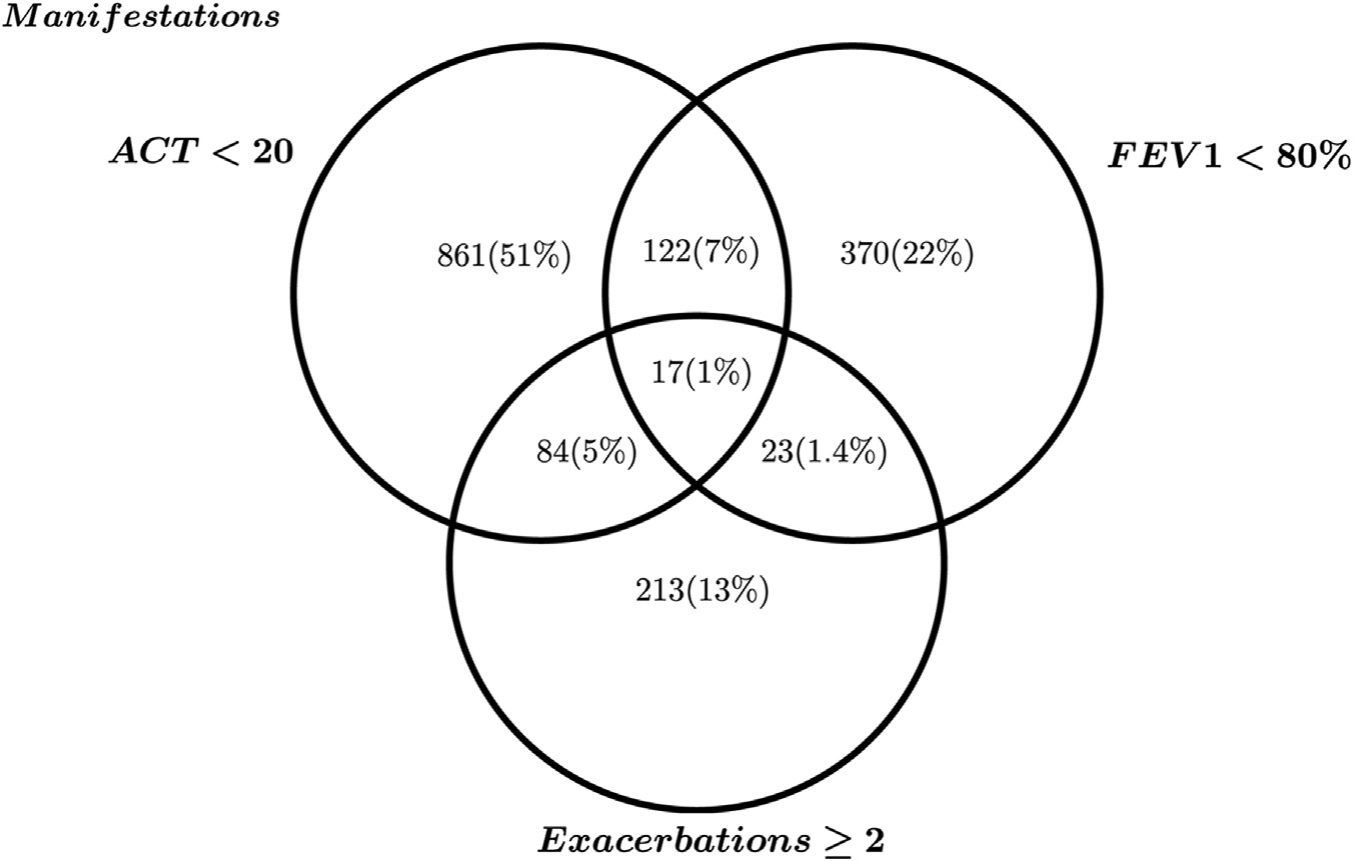
Venn diagram showing the relationship between the 3 different manifestations of UCA in this cohort.

Among the children with UCA (n = 1690), an ACT score of 19 or lower was the most common manifestation (64%) followed by an FEV_1_ value less than 80% predicted (31%) and recurrent exacerbations (20%) (Fig 2). The majority (85%) had only 1 manifestation of UCA, whereas 14% had 2 manifestations and 1% had all 3 manifestations (Table III).

**Table III:** Basic characteristics of children with asthma by treatment steps

Characteristic	Treatment steps 1 and 2	Treatment step 3	Treatment steps 4 and 5	*P* value
	n = 3162	n = 1608	n = 727	
Percentage of entire cohort	57.5%	29.3%	13.2%	
Female sex, no. (%)	1197 (37.9)	632 (39.3)	262 (36.0)	.306
Allergy, no. (%)	1500 (47.4)	852 (53.0)	406 (55.8)	**<.001**
Normal weight, no. (%)	2288 (77.7)	1172 (76.9)	492 (73.3)	
Overweight, no. (%)	509 (17.3)	261 (17.1)	128 (19.1)	
Obesity, no. (%)	146 (5.0)	91 (6.0)	51 (7.6)	**.043**
ACT score, mean (SD)	21.6 (3.6)	21.0 (3.6)	20.5 (3.9)	**<.001**
ACT score ≤ 19, no. (%)	527 (21.7)	368 (27.8)	189 (32.4)	**<.001**
≥2 exacerbations, no. (%)	215 (6.8)	85 (5.3)	37 (5.1)	.054
FEV_1_% predicted, mean (SD)	91.5 (12.0)	90.9 (12.9)	91.5 (13.2)	.339
FEV_1_ < 80% predicted, no. (%)	279 (14.5)	183 (17.3)	70 (15.4)	.123
UCA, no. (%)∗	887 (28.1)	551 (34.3)	252 (34.7)	**<.001**
0-3 factors for UCA†				
0	2275 (71.9)	1057 (65.7)	475 (65.3)	
1	763 (24.1)	470 (29.2)	211 (29.0)	
2	114 (3.6)	77 (4.8)	38 (5.2)	
3	10 (0.3)	4 (0.2)	3 (0.4)	**<.001**

Percentages and mean values calculated for those with complete data regarding BMI, ACT score, and FEV_1_ value. Boldface indicates statistical significance.

∗ UCA defined as an ACT score of 19 or lower and/or 2 or more exacerbations in the past year and/or an FEV_1_ value less than 80% predicted.

† Between 1 and 3 of the following factors: ACT score of 19 lower and/or fewer than 2 exacerbations in th4 past year and/or an FEV_1_ value less than 80% of predicted.

### Pharmacologic treatment

The majority of children were prescribed GINA steps 1 and 2 treatment (58%), followed by step 3 (29%) and steps 4 and 5 (13%) (Fig 1, *B* and Table III). Allergy and obesity were most common among children receiving steps 4 and 5 treatment, whereas the distribution of girls and boys was similar at each treatment step. UCA was common in children receiving all treatment steps, but it was most prevalent among children prescribed steps 4 and 5 treatment (group UCA4-5) (34.7%). The proportion of children with more than 1 manifestation of UCA was highest in children receiving steps 4 and 5 treatment.

### Asthma control in children prescribed steps 1 and 2 treatment

Among all children prescribed steps 1 and 2 treatment, 28% (n = 887) had UCA (group UCA1-2) (Table III). The children in group UCA1-2 were older and more frequently female and obese than those with controlled asthma who were prescribed steps 1 and 2 treatment (Table IV). In adjusted regression analysis, age and female sex remained significant (Table II).

**Table IV:** Basic characteristics of children with UCA and controlled asthma who were receiving steps 1 and 2 treatment

Characteristic	UCA steps 1 and 2	Controlled asthma steps 1 and 2	*P* value
	n = 887	n = 2275	
Female sex, no. (%)	378 (42.6)	819 (36.0)	**<.001**
Age (y), mean (SD)	10.9 (3.1)	10.4 (3.1)	**<.001**
Age 6-11 y, no. (%)	518 (58.4)	1464 (64.4)	
Age 12-17 y, no. (%)	369 (41.6)	811 (35.6)	**.001**
BMI (kg/m^2^), mean (SD)	19.0 (3.8)	18.7 (3.6)	.020
Normal weight, no. (%)	659 (75.9)	1629 (78.5)	
Overweight, no. (%)	160 (18.4)	349 (16.8)	
Obesity, no. (%)	49 (5.6)	97 (4.7)	.271
Allergy, no. (%)	434 (48.9)	1066 (46.9)	.295
ACT score, mean (SD)	18.7 (4.2)	23.1 (1.9)	**<.001**
≥2 exacerbations, no. (%)	215 (24.2)	0 (0.0)	NA
FEV_1_% predicted, mean (SD)	85.9 (13.8)	94.7 (9.6)	**<.001**
Treatment plan, no. (%)	416 (46.9)	865 (38.0)	**<.001**
Asthma management education, no. (%)	644 (72.6)	1407 (61.8)	**<.001**

UCA defined as an ACT score of 19 or lower and/or 2 or more exacerbations in the past year ≥2 and/or an FEV_1_ value less than 80% predicted. Percentages and mean values calculated for those with complete data on BMI, ACT score, and FEV_1_ value. Boldface indicates statistical significance.

### UCA in children prescribed steps 1 and 2 treatment versus steps 4 and 5 treatment

The children in group UCA1-2 were younger, were less often obese, had an allergy less often, had a higher ACT score, and experienced more exacerbations than did children in group UCA4-5 (Table V). The 2 groups did not differ in terms of FEV_1_% value or treatment plan, but the children in group UCA4-5 received asthma management education more frequently (Table V).

**Table V:** Basic characteristics of children with UCA who were receiving step 1 and step 2 treatment versus those of children with UCA prescribed step 4 and step 5 treatment

Characteristic	Uncontrolled steps 1 and 2	Uncontrolled steps 4 and 5	*P* value
	n = 887	n = 252	
Female sex, no. (%)	378 (42.6)	106 (42.1)	.876
Age (y), mean (SD)	10.9 (3.1)	12.4 (2.9)	**<.001**
Age 6-11 y, no. (%)	518 (58.4)	91 (36.1)	
Age 12-17 y, no. (%)	369 (41.6)	161 (63.9)	**<.001**
BMI (kg/m^2^), mean (SD)	19.0 (3.8)	20.7 (4.6)	**<.001**
Normal weight, no. (%)	659 (75.9)	176 (71.8)	
Overweight, no. (%)	160 (18.4)	43 (17.6)	
Obesity, no. (%)	49 (5.6)	26 (10.6)	**.023**
Allergy, no. (%)	434 (48.9)	148 (58.7)	.006
ACT, mean (SD)	18.7 (4.2)	17.2 (3.9)	**<.001**
≥2 exacerbations, no. (%)	215 (24.2)	37 (14.7)	**.001**
FEV_1_% predicted, mean (SD)	85.9 (13.8)	86.6 (15.1)	.299
Treatment plan, no. (%)	416 (46.9)	113 (44.8)	.563
Asthma management education, no. (%)	644 (72.6)	199 (79.0)	.042

UCA defined as an ACT score of 19 or lower and/or 2 or more exacerbations in the past year and/or an FEV_1_ value less than 80% predicted. Percentages and mean values calculated for those with complete data on BMI, ACT score, and FEV_1_ value. Boldface indicates statistical significance.

## Discussion

The goal of asthma management is asthma control, which includes good control of symptoms and minimization of the risk of exacerbations and persistent airflow limitation.^6^ In the current sizeable, real-life study of 5497 school-aged children with asthma, we investigated prevalence and characteristics of UCA as well as associations with prescribed treatment. An important finding was that 31% of the children studied had UCA, which occurred across all ranges of prescribed treatment. Furthermore, we found that female sex, increasing age, obesity, and higher GINA treatment step were associated with UCA. Finally, we found that 28% of the children prescribed steps 1 and 2 treatment had UCA and that exacerbations were more common in this group than in the children with UCA who were prescribed steps 4 and 5 treatment.

Children prescribed less than maximum treatment and having an uncontrolled disease could be considered undertreated patients. Explanations for the seemingly high number of undertreated children include new referrals to specialist care, the variable clinical course of asthma, poor adherence, and the fact that nonpharmacologic treatment alternatives must be evaluated before pharmacologic treatment is intensified. Of note, any benefits of escalation of controller medication may be evident after 3 to 4 months, and once asthma control has been achieved and maintained for 2 to 3 months treatment can often be reduced again.^6^

A few older studies using different classifications of severity and control have reported a prevalence of 60% to 80% of undertreated asthma in adults and children.^12^, ^13^, ^14^ Our findings confirm and extend these older findings by including clinical data from a population-based cohort and using updated and established guidelines for the classification of asthma control.

Undertreatment poses an increased disease burden on the individual patient, including both short-term risks such as increased symptoms and greater need for health care resources, as well as long-term risks, including a decline in lung function.^15^ In a Swedish register–based study, overuse of SABAs (≥3 SABA canisters per year), which are considered a proxy for undertreatment with ICS, was associated with increased risk of exacerbations.^16^ Accordingly, it is reasonable to assume that the number of patients undertreated at any time should be kept as low as possible. Undertreated asthma is possible to identify according to current international classifications of severity and control. Drivers for adjusting treatment in children have recently been investigated in a tertiary care setting, and female sex, poor asthma control, and lower FEV_1_ value were associated with stepping up treatment.^17^

The proportion of undertreated children in any asthma cohort or clinic could be considered a marker of the quality of care provided to asthmatic patients. The concept of undertreated asthma is particularly relevant, as international guidelines have recently or will be revised toward the prescription of an ICS plus a LABA as needed as steps 1 and 2 treatment.^18^ Longitudinal studies will reveal whether these changes will reduce the number of children with undertreated asthma.

A reduced ACT score (64%) was the most common clinical manifestation of UCA and was found more commonly in females. This sex difference in asthma control has been demonstrated previously in adults^19^ and adolescents^20^ and is suggested to be caused by sex-specific physiologic differences and sex-specific behavioral differences.^21^ Standardized questionnaires such as the ACT are useful tools to assess asthma symptoms. Still, the ACT is insufficient for a complete assessment of asthma control, as exacerbations and pulmonary function are not included. This is an essential limitation, as the current findings demonstrate that UCA can be manifested by recurrent exacerbations and/or reduced pulmonary function in a significant proportion of children with normal scores on the ACT. In addition, daytime and or nighttime symptoms can be controlled by a LABA alone, leaving the patient at risk for severe exacerbations, as the underlying inflammation is not treated.^22^ It should also be emphasized that low ACT scores may have explanations other than asthma, including lack of fitness, rhinitis, exercise-induced laryngeal obstruction, and anxiety.^6^

Airflow limitation was the second most common manifestation of UCA (31%), and males had the lowest mean FEV_1_ value. Childhood asthma attenuates the development of lung function throughout adolescence and adulthood,^23^ and our findings are in line with those of previous studies, which have shown that male sex is a risk factor for lower lung function.^15^ In all, 23% of the children with UCA in our cohort had reduced FEV_1_ values but normal ACT scores, a phenomenon that can be explained by poor perception of symptoms^24^ caused by untreated airway inflammation.^25^

Although lung function measurements correlate poorly with chronic asthma symptoms^26^ and are poor discriminators of asthma severity,^27^^,^^28^ it should be kept in mind that a low FEV_1_ value is, together with allergic sensitization and previous exacerbations, a strong predictor of recurrent exacerbations.^29^ The different manifestations of UCA are important, as they might affect the treatment prescribed; a child who does not report symptoms but has a fixed airflow limitation might not be perceived as sick as a child who experiences daily symptoms. In addition, differential diagnoses, such as restrictive lung disease, should be considered in children with normal scores on the ACT and no exacerbations.

Recurrent exacerbations occurred in 6% of the children in the entire cohort and 20% of those with UCA. Exacerbations are not always preceded by poor symptom control, and in the current study, recurrent exacerbations were the sole manifestation of UCA in 11% of participants. It is noteworthy that children prescribed steps 1 and 2 treatment had more exacerbations than did those prescribed steps 4 and 5 treatment. This finding indicates that even though they are an important predictor of future exacerbations, previous exacerbations are not properly taken into account when creating treatment plans for patients.

Our study adds to the limited data on the clinical manifestations of UCA in children. Its strengths include the size of this pediatric specialist care cohort and detailed assessments of asthma control using standardized questionnaires, including the ACT and pulmonary function measurement). The inclusion of patients nationwide reduces selection bias and increases the generalizability of results. The study’s limitations include the fact that we do not know whether the recordings in the SNAR originate from new referrals or follow-up visits, the fact that no data on the dosage of medication are available, the fact that the definition of exacerbations is not uniform, and the fact that registry-based data on the prescription of medications do not reflect actual medication use. Dropout analysis supports the idea that missing data regarding ACT score and FEV_1_ value did not affect the results, as most of the dropouts were younger children with mild asthma and few exacerbations.

In summary, almost one-third of school-aged children treated in specialist care had UCA. Increasing age, female sex, obesity, and higher GINA treatment step were associated with UCA. UCA was common in children prescribed less than the maximum treatment, and those children could be considered undertreated patients. If such children remain undertreated following an evaluation and adjustment of deteriorating factors, inhalation technique, and adherence, clinicians should intensify pharmacologic treatment. We suggest that the proportion of undertreated children in any asthma cohort or clinic might be a useful marker of the quality of care provided to those patients.

## Disclosure statement

Supported by the 10.13039/501100003793Swedish Heart-Lung Foundation (grant 20200548 [to C.S.]) and Region Norrbotten (grant to C.S.), Region Stockholm (a scholarship to J.R.K.), the Freemason Child House Foundation (a grant to J.R.K.), the Konsul Th. C. Bergh’s Foundation (a grant to J.R.K.), the Swedish Asthma and Allergy Association’s Research Foundation (a grant to J.R.K.), the 10.13039/501100003793Swedish Heart-Lung Foundation (a grant to J.R.K.), and the Pediatric Research Foundation of Astrid Lindgren Children’s Hospital (a grant to J.R.K.).

Disclosure of potential conflict of interest: C. Stridsman reports personal fees from 10.13039/100004325AstraZeneca, Boehringer Ingelheim, and Novartis outside the submitted work. J. R. Konradsen reports personal fees from 10.13039/100004336Novartis and institutional fees from 10.13039/100009857Regeneron Pharmaceuticals and 10.13039/100011033Thermo Fisher Scientific outside the submitted work. The rest of the authors declare that they have no relevant conflicts of interest.

## Supplementary data

Supplementary Table E1

## References

[1] Porsbjerg C., Melen E., Lehtimaki L., Shaw D. (2023). Asthma. Lancet.

[2] Chung K.F., Wenzel S.E., Brozek J.L., Bush A., Castro M., Sterk P.J. (2014). International ERS/ATS guidelines on definition, evaluation and treatment of severe asthma. Eur Respir J.

[3] Shipp C.L., Gergen P.J., Gern J.E., Matsui E.C., Guilbert T.W. (2023). Asthma management in children. J Allergy Clin Immunol Pract.

[4] Dinakar C., Chipps B.E. (2017). Section on Allergy and Immunology, Section on Pediatric Pulmonology and Sleep Medicine. Clinical tools to assess asthma control in children. Pediatrics.

[5] Papadopoulos N.G., Arakawa H., Carlsen K.H., Custovic A., Gern J., Lemanske R. (2012). International consensus on (ICON) pediatric asthma. Allergy.

[6] GINA Global Initiative for Asthma From the Global Strategy for Asthma Management and Prevention 2022. https://ginasthma.org/wp-content/uploads/2023/07/GINA-2023-Full-report-23_07_06-WMS.pdf/.

[7] Garcia-Marcos L., Chiang C.Y., Asher M.I., Marks G.B., El Sony A., Masekela R. (2023). Asthma management and control in children, adolescents, and adults in 25 countries: a Global Asthma Network Phase I cross-sectional study. Lancet Glob Health.

[8] Stridsman C., Konradsen J.R., Vanfleteren L., Pedroletti C., Binnmyr J., Edfelt P. (2020). The Swedish National Airway Register (SNAR): development, design and utility to date. Eur Clin Respir J.

[9] Boyle R.J., Pedroletti C., Wickman M., Bjermer L., Valovirta E., Dahl R. (2012). Nocturnal temperature controlled laminar airflow for treating atopic asthma: a randomised controlled trial. Thorax.

[10] Cole T.J., Lobstein T. (2012). Extended international (IOTF) body mass index cut-offs for thinness, overweight and obesity. Pediatr Obes.

[11] Solymar L., Aronsson P.H., Bake B., Bjure J. (1980). Nitrogen single breath test, flow-volume curves and spirometry in healthy children, 7-18 years of age. Eur J Respir Dis.

[12] Nolte H., Nepper-Christensen S., Backer V. (2006). Unawareness and undertreatment of asthma and allergic rhinitis in a general population. Respir Med.

[13] Ofoma U.R., Lehman E., C S (2012). Undertreated and uncontrolled asthma among us adults: findings from a national sample. Am J Respir Crit Care Med.

[14] Fuhrman C., Dubus J.C., Marguet C., Delacourt C., Thumerelle C., de Blic J. (2011). Hospitalizations for asthma in children are linked to undertreatment and insufficient asthma education. J Asthma.

[15] McGeachie M.J., Yates K.P., Zhou X., Guo F., Sternberg A.L., Van Natta M.L. (2016). Patterns of growth and decline in lung function in persistent childhood asthma. N Engl J Med.

[16] Melen E., Nwaru B.I., Wiklund F., de Fine Licht S., Telg G., Maslova E. (2022). Short-acting beta(2) -agonist use and asthma exacerbations in Swedish children: a SABINA junior study. Pediatr Allergy Immunol.

[17] Ardura-Garcia C., Pedersen E.S.L., Mallet M.C., de Jong C.C.M., Barben J., Jochmann A., Jung A. (2022). Treatment decisions in children with asthma in a real-life clinical setting: the Swiss Paediatric Airway Cohort. J Allergy Clin Immunol Pract.

[18] Bush A., Holguin F., Porsbjerg C., Saglani S. (2023). Asthma: closing in on the biology of a complex life-course disease. Am J Respir Crit Care Med.

[19] Colombo D., Zagni E., Ferri F., Canonica G.W., PROXIMA Study Centers (2019). Gender differences in asthma perception and its impact on quality of life: a post hoc analysis of the PROXIMA (Patient reported outcomes and Xolair) in the management of asthma) study. Allergy Asthma Clin Immunol.

[20] Stridsman C., Backman H., Eklund B.M., Ronmark E., Hedman L. (2017). Adolescent girls with asthma have worse asthma control and health-related quality of life than boys-a population based study. Pediatr Pulmonol.

[21] Boulet L.P., Lavoie K.L., Raherison-Semjen C., Kaplan A., Singh D., Jenkins C.R. (2022). Addressing sex and gender to improve asthma management. NPJ Prim Care Respir Med.

[22] Lazarus S.C., Boushey H.A., Fahy J.V., Chinchilli V.M., Lemanske R.F., Sorkness C.A. (2001). Long-acting beta2-agonist monotherapy vs continued therapy with inhaled corticosteroids in patients with persistent asthma: a randomized controlled trial. JAMA.

[23] Odling M., Wang G., Andersson N., Hallberg J., Janson C., Bergstrom A. (2021). Characterization of asthma trajectories from infancy to young adulthood. J Allergy Clin Immunol Pract.

[24] Sutherland T.J., Cowan J.O., Taylor D.R. (2008). Dynamic hyperinflation with bronchoconstriction: differences between obese and nonobese women with asthma. Am J Respir Crit Care Med.

[25] Barnes P.J., Szefler S.J., Reddel H.K., Chipps B.E. (2019). Symptoms and perception of airway obstruction in asthmatic patients: clinical implications for use of reliever medications. J Allergy Clin Immunol.

[26] Chu F., Kappel N., Akel M., Press V.G., Alexander J.T., Volerman A. (2023). Validity of the childhood asthma control test in diverse populations: a systematic review. Pediatr Pulmonol.

[27] Gaffin J.M., Petty C.R., Sorkness R.L., Denlinger L.C., Phillips B.R., Ly N.P. (2023). Determinants of lung function across childhood in the Severe Asthma Research Program (SARP) 3. J Allergy Clin Immunol.

[28] Lang A.M., Konradsen J., Carlsen K.H., Sachs-Olsen C., Mowinckel P., Hedlin G. (2010). Identifying problematic severe asthma in the individual child--does lung function matter?. Acta Paediatr.

[29] Grunwell J.R., Gillespie S., Morris C.R., Fitzpatrick A.M. (2020). Latent class analysis of school-age children at risk for asthma exacerbation. J Allergy Clin Immunol Pract.

