# Supplementary material for: Uncontrolled asthma in school-aged children—a nationwide specialist care study
Source: J Allergy Clin Immunol Glob. 2024 Feb 13;3(2):100227. doi: 10.1016/j.jacig.2024.100227 (PMC10910119; doi:10.1016/j.jacig.2024.100227)
Supplement: Supplementary Table E1 [file mmc1.docx]

|  | **ACT** | **Missing ACT** |  | **FEV_1_** | **Missing FEV_1_** |  |
| --- | --- | --- | --- | --- | --- | --- |
|  | **n=4335** | **n=1162** | **P-value** | **n=3435** | **n=2062** | **p-value** |
| **Age, mean (sd)** | 11.4 (3.1) | 10.1 (3.3) | <0.001 | 11.4 (3.1) | 10.8 (3.5) | <0.001 |
| **Females, n (%)** | 1634 (37.7) | 457 (39.3) | 0.308 | 1324 (38.5) | 767 (37.2) | 0.319 |
| **BMI, mean (sd)** | 19.5 (3.9) | 18.6 (3.8) | <0.001 | 19.4 (3.9) | 19.1 (3.9) | 0.001 |
| **Exacerbations ≥2, n (%)** | 294 (6.8) | 43 (3.7) | <0.001 | 224 (6.5) | 113 (5.5) | 0.119 |
| **Step 1-2, n (%)** | 2428 (56.0) | 734 (63.2) |  | 1925 (56.0) | 1237 (60.0) |  |
| **Step 3, n (%)** | 1323 (30.5) | 285 (24.5) |  | 1056 (30.7) | 552 (26.8) |  |
| **Step 4-5, n (%)** | 584 (13.5) | 143 (12.3) | <0.001 | 454 (13.2) | 273 (13.2) | 0.005 |
| **FEV_1_% predicted, mean (sd)** | 91.4 (12.5) | 90.7 (12.2) | 0.148 |  |  |  |
| **ACT, mean (sd)** |  |  |  | 21.2 (3.7) | 21.4 (3.5) | 0.069 |

BMI=Body Mass Index. FEV_1_= Forced Expiratory Volume in one second. ACT=Asthma Control Test.
